# Exploring the relationship between air quality and health shocks to the elderly: A retrospective cross-sectional study in China

**DOI:** 10.3389/fpubh.2023.1087626

**Published:** 2023-03-27

**Authors:** Xinyu Li, Yanxia Lyu, Wanyue Dong, Aijun Xu

**Affiliations:** ^1^School of Health Economics and Management, Nanjing University of Chinese Medicine, Nanjing, China; ^2^Jiangsu Research Center for Major Health Risk Management and TCM Control Policy, Nanjing University of Chinese Medicine, Nanjing, China

**Keywords:** air pollution, health shocks, the elderly, self-rated health (SRH), activities of daily living

## Abstract

**Methods:**

We selected 5,172 microdata on individuals from the China Health and Retirement Longitudinal Study (CHARLS) 2018. The binary logit model, the ordered logit model, and the stepwise regression were employed to compare the effects of air pollution on self-rated health (SRH) and activities of daily living (ADL) in an elderly population. The effects on health shocks were explored in different age groups, different gender groups, different regions and different sources of pollutants, respectively.

**Results:**

We found that air pollution significantly increased the risk of health shocks in the elderly population, especially in the 60–69 year age group, and the eastern/central region, where NO_2_ and O_3_ were important pollutant sources.

**Conclusion:**

Targeted management of the environment is necessary to improve the health status of China's elderly population. In addition, paying attention to the health status of vulnerable populations is needed to achieve social equity.

## 1. Introduction

Haze is a major manifestation of air pollution (AP) that influences most urban areas of China, especially in the northern regions. Respirable particulate matter in haze contains various chemicals, such as sulfur dioxide, metal elements and radioactive substances, which have a major impact on human health (1). A recent study published by the World Health Organization (WHO) in 2018 states that ~7 million people die globally each year as a result of exposure to fine particles in polluted air. In its 2022 air quality database update, WHO states that almost the entire global population (99%) breathes air that contains high levels of pollutants; air quality has already passed beyond the limit of the WHO guideline and pollution is 90% higher than it was 4 years ago (2). The report “Toward an Environmentally Sustainable Future” also states that <1% of China's 500 largest cities meet the WHO recommended air quality standards (3).

Moreover, with the development of China's economy, the standard of living and health of the people have improved significantly, and the life expectancy of the elderly population has increased. According to China's seventh National Census, the population will total 1,411.78 million in 2021. The elderly population, aged 65 and above, is 190.64 million, which accounts for 13.5% of the total population, and has increased by 4.63 percentage points compared with the sixth National Census in 2010 (4). According to the prevailing international standards for judging aging societies, China has long since become an aging society. When compared with developed countries, China's aging population has several characteristics, such as a large and rapidly increasing number of elderly people (5). Older people are usually in poorer health, with deep levels of multiple morbidity and chronic diseases (6). The elderly population is chronically exposed to diseases, and air pollution is an important environmental risk factor that has an impact on both physical and mental health. Older people have been exposed to poor air quality over many years, accompanied by a decline in physical function and resistance, loss of health, accelerated depreciation of physical health capital in the face of air pollution, and a greater likelihood of disease impact (7–9). Despite advances in medical technology, increased national investment in health over the years, and updated air governance policies, older people are still at serious risk of health shocks and tend to have a greater prevalence of comorbidities (10), which already pose a challenge to the public health service system.

The level of air quality reflects the degree of AP, which is affected by the type and concentration of pollutants in the air. Currently, the Air Quality Index (AQI), the concentration of sulfur dioxide (SO_2_), nitrogen dioxide (NO_2_), inhalable particles (PM_10_), ozone (O_3_), respirable particulate matter (PM_10_) and fine particulate matter (PM_2.5_) are the primary indicators used to assess air quality (11, 12). Group differences have been confirmed by research on population health and air quality, and the elderly are particularly vulnerable to the adverse effects of air pollution (13, 14). Hence, some studies have specifically addressed health issues in older populations and found that air pollution has negative effects on health status and mental health (15–17), significantly increasing the probability of ADL disability (18, 19). However, in an aging society, the sudden deterioration of health conditions suffered by the senior population should be more of a public concern. Previous research using microdata has mostly concentrated on positive health scores or disability. This study introduces the variable of health shock and attempts to combine previous studies to measure the impact of exposure in terms of deterioration in self-rated health and the ability to perform daily behaviors.

Based on the above research background, we investigated the extent to which AP affected the health impacts of older people, using data from the air quality historical data query platform and the China Health and Retirement Longitudinal Study (CHARLS) 2018, selecting people aged 60 years and older as the study population.

## 2. Materials and methods

### 2.1. Study population

The micro-data used in this paper came from the China Health and Retirement Tracking Survey, a national baseline of the China Health and Retirement Longitudinal Study (CHARLS), organized by the National Development Institute of Peking University and launched in 2011. CHARLS data focused on middle-aged and older people, aged 45 and above, and were updated to the 2018 sample survey data. In sampling, CHARLS used a stratified (personal GDP by urban area and rural county) multi-stage (county/district, village/community, households) random sampling method proportional to population size (PPS), with strict control of sample quality, so that the data represented the overall situation in China. In this paper, people aged 60 years and older were selected as the study population. In addition to being easily exposed to AP, this group has the traits of rarely moving their residences and hardly changing their socio-economic status (11). Therefore, it is easier to explore the correlation by analyzing the elderly population.

AP can be divided into two categories: indoor AP and outdoor AP (12). Owing to the differences in the sources of major pollutants in different regions of China and at different times in the same region, we used data on six common air pollutant indicators in Chinese cities and selected the air quality composite indicator as a proxy variable for AP in each region. The AQI, which is calculated based on hourly concentration readings of the above six categories of pollutants, is a reliable indicator for quantitative assessment of health risks (20, 21). The China Ambient Air Quality Standard (CAAQS) (GB 3095-2012) was implemented in 2012 after the Chinese Ministry of Environmental Protection (CMEP) announced the Ambient Air Quality Index Technical Provisions (Trial) (HJ 633-2012) (22, 23). The higher the AQI value, the more serious the AP (22). AP data were obtained from the air quality historical data query platform, which provides a rich and reliable data source for AP-related research (24–26). Considering that there may be a certain lag in the effect of AP on individual health shocks, we selected the average value of 2017–2018 to examine the relationship between air quality and individuals' loss of independent living due to physical dysfunction and to explore what caused the loss of health in older people. Monthly air quality data (including date, AQI, range, quality class, PM_2.5_, PM_10_, SO_2_, NO_2_, CO, O_3_, and city) for 2017 and 2018 for nearly 400 cities in China were crawled using PYTHON 3.9.7. To ensure the quality of our data, we selected PM_2.5_ in 2018 from another database (27) and proofread the data with the PM_2.5_ obtained in our study. The average values are 39.541 μg/m^3^ in the Historical Data Platform and 39.217 μg/m^3^ in the Data Center of Ministry of Ecology and Environment; therefore it can be considered that our air quality data are reliable. The 2018 China Statistical Yearbook's urban and environmental chapters provided the city-level data (28), and a few missing data were supplemented with provincial data. The database used in this paper was made available to the academic community with the approval of the Peking University Ethics Committee; air quality data and city-level data were public and therefore did not require ethical approval.

This paper first matched the PSU (province, city, name, and area type) sub-data from the CHARLS survey (containing information on an individual's province, city name, and area type) with 2018 personal information to locate the city where the respondent was located, and then used the city name information to match the individual micro-data with the AP data. In the end, 123 cities were successfully matched, and a total of 5,172 samples were obtained after selecting people aged 60 years and above.

### 2.2. Variable selection

#### 2.2.1. Definition of healthy outcome

Health shocks, as a complex concept, are not agreed upon in existing research. Most studies have used changes in self-rated health status (SRH) to measure health shocks: a worse SRH than the previous year or a sharp deterioration in SRH is considered a health shock (29, 30). More recently, researchers have considered limitations in daily activities as a direct indicator of health shocks (31, 32). The term “health shock” was used in this paper to refer to negative health events associated with the loss of an individual's ability to live independently due to physical impairment and was measured by two indicators, a subjective self-assessment of respondent health, SRH (29), and an objective self-assessment of the respondent's ability to perform activities of daily living (31). The CHARLS questionnaire's SRH was measured by asking the respondents “How do you think your health is?” The question was divided into five levels: “very good,” “good,” “fair,” “poor” and “very poor.”

Good functional status is fundamental to maintaining the independence of older people. Objectively, older people are prone to a variety of illnesses that can impair their health, which can lead to a decline in their ability to perform activities of daily living (ADL) (33). The assessment of ADL can provide a basis for diagnosing illnesses, predicting the needs of social services for the elderly, developing treatment plans, and rationalizing the placement of the elderly (34). ADL include Basic Activities of Daily Living (BADL) and Instrumental Activities of Daily Living (IADL), and this paper selected BADL to better reflect the impact of negative health events on the respondents (35). The ADL scale in the CHARLS questionnaire asked respondents whether they had difficulties in daily life, such as dressing and bathing.

#### 2.2.2. Exposure assessment for air quality index

Fine particulate air pollution <2.5 μm diameter (PM_2.5_) is only one of the main sources of AP and an air quality index that takes into account all sources of pollutants is a more appropriate proxy variable for air pollution (20, 21). The range of the AQI is 0 to 500. The pollutant with the highest concentration is regarded as the primary pollutant when the AQI is higher than 50. We took the annual average value of AQI of the area where the senior population was located as the proxy indicator to reflect long-term AP. Additionally, because the data quality is a major concern, the independent variable AQI was replaced with six single indicators in our paper's robustness check (13).

#### 2.2.3. Measurement of control variables

Referring to Grossman's health needs theory and Zeng et al. (37) regarding the delineation of control variables, and modified to fit the needs of the study, the individual control variables were divided into three categories: demographic characteristics, enabling variables, and social welfare (36, 37). Demographic characteristics included sex, age, marital status, education level, and type of residence. Enabling factors are the tendency for this group to acquire health shocks more than others, a tendency that can be predicted from pre-onset personal characteristics, such as smoking, drinking and a history of present illness (HPI). Social benefits were more focused on pensions for those over 60 years of age. Marital status was reclassified according to the six options in the questionnaire: married (including cohabiting and temporarily not living with spouse due to work or other reasons) and unmarried (including separated, divorced, widowed, never married). The level of education was divided into four categories: (1) primary school and below; (2) junior high school; (3) senior high school; and (4) college or above. Respondents were classified as living in a town if in the center of the city/town or the combination zone between urban and rural areas, with the remainder in villages. Smoking and alcohol consumption, as unhealthy lifestyles, have health consequences, and participating groups are more likely to experience health shocks (29), so we considered “whether they smoke” and “whether they drink” as a control variable. Many older people in China share the responsibility of caring for their grandchildren, and pensions are the main source of socio-economic support for older people with chronic diseases in the face of shocks (35). Older people, therefore, are better able to withstand the threat of health shocks if they have health benefits such as pensions. The CHARLS questionnaire asked respondents “Do you currently receive, expect to receive or contribute to the pension for public servants, or pension for public institution employees, or basic pension for enterprise employees?” to define whether the respondent has a pension. Considering that pre-existing diseases may affect the reliability of the results; we selected three diseases most closely related to AP (38–41). The CHARLS questionnaire asked respondents “Have you been diagnosed with chronic lung diseases, stroke or asthma by a doctor?” to define whether the respondent had HPI.

When measuring the relationship between environmental quality and public health, it is necessary to take into account the overall effect as comprehensively as possible. This paper selected two urban macro variables to control the intensity of regional environmental regulation (42, 43). The water quality was determined by the sewage treatment rate and the green space was measured by the green coverage rate of the built-up area.

### 2.3. Statistical analysis

Generally, linear regression and ordered logit regression are used to study SRH. We used logit regression to judge health status more directly. Herein, ordered logit regression was conducted based on the results of the questionnaire survey. Respondents were assigned a value of “2” if their health status was poor or very poor; “1” if their health status was fair; “0” if their health status was good or very good. The model was


(1) $$\begin{array}{l} H_{ij}\;*\;=\alpha +\beta_{1}\;*\;{\text{Aqi}}_{ij}+\beta_{2}\;*\;Control_{ij}+\varepsilon_{ij} \end{array}$$

Where H represents the micro-individual SRH index, Aqi represents the AQI, Control represents the control variable and ε represents the random perturbation term, i denotes the micro-individual, j denotes the province in which the micro-individual i is located, α denotes the value of the surrogate estimated parameter variable, and β_1_, β_2_ denote the effect of AP and other control variables on the health status of individuals.

Based on ADL competency according to the questionnaire items, respondents were marked as “1” if they met any of the above criteria, or as “0” if no symptoms occurred, and a binary logit regression model was constructed.


(2) $$\begin{array}{l} ln\left(\frac{\rho }{1-\rho }\right)=\partial +\gamma_{1}\;*\;{\text{Aqi}}_{ij}+\gamma_{2}\;*\;Control_{ij\;*}+\varepsilon_{ij^{*}} \end{array}$$

Where ρ represents the probability that ADL = 1 and “1–ρ” represents the probability that ADL = 0. In this study, the stepwise regression method was used to introduce variables into the model one by one to ensure that only significant variables were included in the regression equation before each new variable was introduced. All statistical procedures in this paper were implemented by STATA 17.0.

## 3. Results

### 3.1. Descriptive statistics of analysis samples

Overall, women accounted for the vast majority of respondents, 79% of the total sample. The highest age was 98 years, with 62.35% of the population between 60 and 69 years old and over 8% of the total population aged over 80 years. Owing to the low level of education in China before the 1970's, more than 76% of the elderly had an education level below junior high school. In terms of place of residence, more than 74% of respondents were from rural areas and only a small proportion had a pension; 20% of the total population had a very good or good SRH status and 32% had a poor or very poor one. It is pleasing to note that only 18% of seniors had an ADL shock, indicating that most seniors can take care of themselves in daily life. At the same time, 6.38% of seniors smoked and 18.97% drank alcohol, indicating that most seniors adopt a healthy lifestyle. Overall, 12.88% of seniors suffered from lung-related diseases, stroke, or asthma. In terms of air quality, the average AQI value was between 77 and 78. 78% of people were living in an environment with an AQI between 50 and 100, with good air quality. However, the results of 5,172 samples reveal that there is a significant difference in air quality levels among cities, with the maximum AQI being three times higher than the minimum. Obviously, compared with the sewage treatment rate, the green coverage rate is not ideal, and there is a gap between cities. The description of the variables and the results of the survey are shown in Tables 1, 2.

**Table 1:** Statistical description of analysis samples created from CHARLS.

	**Self-rated health**					**Activities of daily living**			
	**Very good/Good**	**Fair**	**Very poor/Poor**			**No difficulty**	**Difficulty**		
	***N*** = **1,038**	***N*** = **2,465**	***N*** = **1,669**	^a^ *χ**2***	***P***	***N*** = **4228**	***N*** = **944**	*^**χ2**^*	***P***
**Age group**				26.7544	0.000			40.2321	0.000
60–69	698	1,563	964			2,551	674		
70+	340	902	705			3,831	220		
**Sex**				38.9079	0.000			204.5593	0.000
Female	768	1,937	1,398			3515	588		
Male	270	528	271			713	356		
**Education**				29.1640	0.000			17.0521	0.001
Primary school	483	1,218	897			2144	454		
Junior high	282	649	446			1,150	227		
Senior high	192	382	232			620	186		
College	81	216	94			314	77		
**Marital**				16.1117	0.000			29.4200	0.000
Single	203	539	431			1,022	151		
Married	835	1,926	1,238			3,206	793		
**Residence**				77.9096	0.000			20.4121	0.000
Village	720	1,752	1,368			3,194	646		
Town	318	713	301			1,034	298		
**Pension**				121.5664	0.000			48.1332	0.000
No	785	1,897	1,493			3,489	686		
Yes	253	568	176			739	258		
**Smoke**				0.0884	0.957			30.9441	0.000
No	970	2,310	1,562			3,996	846		
Yes	68	155	107			232	98		
**Drink**				72.2044	0.000			92.8579	0.000
No	763	1,983	1,445			3,531	660		
Yes	275	482	224			697	284		
**HPI**				239.5507	0.000			58.6268	0.000
No	979	2,207	1,320			3,641	865		
Yes	59	258	349			587	79		

a chi-square test and *p*-value.

**Table 2:** Overall characteristics of ^a^ environmental factors in China (2017-2018).

			**Self-rated health**					**Activities of daily living**	
			**Very good/good**	**Fair**	**Very poor/poor**			**No difficulty**	**Difficulty**
	^d^ **F**	***P***	***N*** = **1,038**	***N*** = **2,465**	***N*** = **1,669**	^e^ ***t***	***P***	***N*** = **4,228**	***N*** = **944**
**Mean** ±**SD**									
AQI	2.22	0.1086	78.590 ± 19.722	78.001 ± 18.816	77.049 ± 20.317	3.29	0.0697	77.580 ± 19.543	78.853 ± 19.281
^c^PM_2.5_(μg /m^3^)	2.12	0.1206	42.872 ± 13.637	42.976 ± 13.286	42.106 ± 14.564	3.60	0.0578	42.502 ± 13.892	43.444 ± 13.274
PM_10_(μg /m^3^)	2.59	0.0752	74.207 ± 24.410	73.157 ± 23.146	72.090 ± 24.562	3.78	0.0519	72.718 ± 23.852	74.389 ± 23.947
SO_2_(μg /m^3^)	3.33	0.0359	16.169 ± 7.980	15.505 ± 7.098	15.932 ± 8.010	0.84	0.3597	15.730 ± 7.623	15.981 ± 7.420
CO (mg /m^3^)	1.93	0.1452	0.917 ± 0.230	0.925 ± 0.225	0.910 ± 0.232	4.76	0.0291	0.915 ± 0.229	0.933 ± 0.226
O_3_(μg /m^3^)	5.03	0.0066	93.962 ± 12.360	92.666 ± 12.084	92.569 ± 12.191	4.18	0.0409	92.731 ± 12.175	93.628 ± 12.210
NO_2_(μg /m^3^)	6.67	0.0013	31.527 ± 9.850	31.526 ± 9.625	30.470 ± 9.767	7.21	0.0073	31.014 ± 9.741	31.955 ± 9.689
^b^Green			41.377 ± 5.772	40.928 ± 6.022	41.433 ± 5.838			41.363 ± 5.588	41.785 ± 6.134
Sewpro			95.785 ± 1.708	95.624 ± 1.692	95.723 ± 1.724			95.670 ± 1.725	95.652 ± 1.624

Mean ± SD: mean ± standard deviation.

a Variables from the air quality historical data query platform and yearbooks are city-level.

b *Sewpro:* Sewage treatment rate (%) = (sewage treatment volume / total sewage discharge) × 100%.

*Green*: Green Ratio (%) = (vertical projected area of green plants / urban area) × 100%.

c PM_2.5_(μg /m^3^), PM_10_(μg /m^3^), SO_2_(μg /m^3^), CO (mg /m^3^), O_3_ (μg /m^3^) and NO_2_ (μg /m^3^) represent the concentration of pollutants in the air.

d Analysis of Variance and *p* value.

e Two-group t-test and *p* value.

### 3.2. Effects of air quality index on health shocks

The results of the ordered logit regression (Table 3) show that the effect of AQI on SRH is significant at the 0.05 level after the inclusion of the relevant control variables, implying that AP has a significant positive effect on the deterioration of SRH among the elderly. Models 1 to 4 are the stepwise test results of AQI and SRH. Model 1 exhibits that, without any control variables, AQI successfully passes the significance test and the estimated coefficient of AQI is 0.26, which is significant at the level of 5%. Individual control variables and urban-level control variables in models 2 to 4 are gradually included as control variables, and AQI still has a significant positive impact on SRH (OR = 1.004; 95% CI:1.001–1.007; *P* < 0.05). Among the control variables, SRH is significantly influenced by age (OR = 0.016; 95% CI: 0.007–0.024; *P* < 0.01), sex (OR = 0.724; 95% CI: 0.623–0.841; *P* < 0.05), residence (OR = 0.796; 95% CI: 0.685–0.926; *P* < 0.01), pension (OR = 0.681; 95% CI: 0.575–0.807; *P* < 0.01), smoking (OR = 1.464; 95% CI: 1.160–1.847; *P* < 0.01), drinking (OR = 0.647; 95% CI: 0.562–0.745; *P* < 0.01). Age, smoking, and HPI (OR = 2.710; 95% CI: 2.307–3.184; *P* < 0.01) have negative effects.

**Table 3:** Odds ratios (95% Confidence Intervals) and regression coefficient of cross-sectional impact of 2-year exposure to air pollution on health shocks among Chinese elderly, CHARLS 2018.

	**Model 1**		**Model 2**		**Model 3**		**Model 4 ORs (95% CI)**	
	**SRH (95%CI)**	**ADL (95%CI)**	**SRH (95%CI)**	**ADL (95%CI)**	**SRH (95%CI)**	**ADL (95%CI)**	**SRH (95%CI)**	**ADL (95%CI)**
^a^AQI	^b^0.260^**^	0.274^*^	0.212^**^	0.318^**^	0.259^**^	0.337^**^	0.256^**^	0.399^**^
	^c^(0.058, 0.463)	(−0.004, 0.551)	(0.007, 0.418)	(0.030, 0.607)	(0.052, 0.465)	(0.043, 0.630)	^d^1.004 (1.001, 1.007)	1.005 (1.001, 1.009)
Age			−0.017^***^	−0.053^***^	−0.017^***^	−0.052^***^	−0.017^***^	−0.052^***^
			(−0.025, −0.009)	(−0.065, −0.040)	(−0.026, −0.009)	(−0.065, −0.039)	0.016 (0.007, 0.024)	0.951 (0.939, 0.963)
Sex			0.406^***^	1.157^***^	0.303^***^	1.031^***^	0.303^***^	1.030^***^
			(0.277, 0.536)	(0.996, 1.318)	(0.153, 0.452)	(0.843, 1.219)	0.724 (0.623, 0.841)	2.811 (2.333, 3.389)
Married			0.114^*^	0.120	0.083	0.103	0.083	0.108
			(−0.018, 0.247)	(−0.082, 0.323)	(−0.051, 0.218)	(−0.102, 0.308)	0.916 (0.801, 1.048)	1.124 (0.917, 1.378)
Resid			0.410^***^	0.237^***^	0.242^***^	0.084	0.242^***^	0.080
			(0.288, 0.533)	(0.070, 0.404)	(0.091, 0.394)	(−0.126, 0.294)	0.796 (0.685, 0.926)	1.067 (0.866, 1.315)
Educa			0.075	−0.052	0.057	−0.072	0.057	−0.069
			(−0.050, 0.200)	(−0.235, 0.130)	(−0.069, 0.184)	(−0.257, 0.114)	(−0.069, 0.184)	(−0.255, 0.116)
			0.218^***^	0.382^***^	0.157^**^	0.342^***^	0.157^**^	0.347^***^
			(0.065, 0.371)	(0.178, 0.587)	(0.002, 0.313)	(0.133, 0.550)	(0.002, 0.313)	(0.139, 0.556)
			0.223^**^	0.191	0.149	0.142	0.149	0.142
			(−0.019, 0.427)	(−0.098, 0.481)	(−0.060, 0.358)	(−0.154, 0.438)	0.935 (0.883, 0.991)	1.101 (1.017, 1.193)
Pension					0.452^***^	0.338^***^	0.452^***^	0.332^***^
					(0.283, 0.621)	(0.112, 0.563)	0.681 (0.575, 0.807)	1.337 (1.069, 1.674)
Smoke					−0.314^***^	−0.031	−0.314^***^	−0.027
					(−0.547, −0.081)	(−0.315, 0.254)	1.464 (1.160, 1.847)	0.913 (0.688, 1.212)
Drink					0.404^***^	0.335^***^	0.404^***^	0.339^***^
					(0.262, 0.545)	(0.154, 0.517)	0.647 (0.562, 0.745)	1.439 (1.202, 1.724)
HPI					−0.866^***^	−0.614^***^	−0.866^***^	−0.612^***^
					(−0.972, −0.759)	(-0.766, −0.463)	2.710 (2.307, 3.184)	0.570 (0.442, 0.735)
Green							−0.001	0.004
							0.981 (0.959, 1.003)	1.021 (0.989, 1.055)
Sewpro							0.001	−0.029
							0.981 (0.959, 1.003)	0.961 (0.917, 1.007)

Marri, Marital status; Resid, Residence; Educa, Education; HPI, history of present illness.

a The AQI were ln-transformed in these models.

b Stepwise regression Coefficient: Standard errors in parentheses, clustered at the Provincial level.

^*^, ^**^, and ^***^ indicate significance at the levels of 10, 5, and 1%, respectively.

c 95% confidence intervals.

d The column of model 4 is the confidence interval of Odds ratios (ORs) (95% CI).

In binary logit regression (Table 3), model 1 demonstrates that the estimated coefficient of AQI is 0.274, which is significant at the level of 10%, in the absence of any control variables. After accounting for all control variables, the estimated coefficient of AQI is 0.399 (OR = 1.005; 95% CI: 1.001–1.009; *P* < 0.05). Similarly, we also found that age (OR = 0.951; 95% CI: 0.939–0.963; *P* < 0.01) has a significant negative impact on ADL.

### 3.3. Heterogeneity analysis between subgroups

#### 3.3.1. Comparative analysis of different ages, sexes, and regions

To analyze the difference in the relationship between air quality and health shocks, heterogeneity tests were conducted based on age, gender, and regional groups. According to the division of age from Hu et al. (11) and Zeng et al. (37), our samples were divided into 60–69 and 70+ years (we classified these two groups as “young-older” and “older,” respectively. The gender groups were divided into male samples and female samples. Regional groups were divided into eastern, central, and western regions according to geographical location. The regression analyses were conducted on these sub-samples to examine how the effects of AP on health shocks in older people differed across age, sex, and regional groups. The effect of AQI on SRH and ADL is significant in the “young-older” group (Table 4). Specifically, the estimated coefficients are 0.1074 for SRH (*P* < 0.05) and 0.0694 (*P* < 0.05) for ADL, respectively. The “older” group did not pass the significance test. Compared with the “older” group, air quality has a significantly greater impact on the “young-older” group. AQI has a significant effect on impaired daily behavior in regions with different levels of economic development, but we did not observe a statistical significance in SRH. Specifically, as shown in Table 5, the estimated coefficient of ADL in the eastern region is 0.1159 (*P* < 0.05); the estimated coefficient of ADL in the central region is 0.2444 (*P* < 0.01). Finally, we conducted interaction effect analyses, the results showed that there were significant differences in age groups (*P* < 0.01) and regional groups (*P* < 0.1), but no significant difference in sex groups (details in the Table 6). Therefore, it can be considered that AQI has a differential impact with regard to health shocks at different age or regional groups.

**Table 4:** Heterogeneity of SRH and ADL among different age groups of residents affected by air quality index.

	**Age > 60**		**60–69**		**70**+		^a^ **Age#AQI**	
	**(1)**		**(2)**		**(3)**		**(4)**	
	**SRH**	**ADL**	**SRH**	**ADL**	**SRH**	**ADL**	**SRH**	**ADL**
AQI	0.0822^**^	0.0534^**^	0.1074^**^	0.0694^**^	0.0558	0.0253	−0.0001	−0.0135^***^
	(0.0410)	(0.0132)	(0.0287)	(0.0119)	(0.4232)	(0.4579)	(0.9472)	(0.0000)
cons	0.4044	0.2960	0.3933	0.0088	0.1614	0.7075		
	(0.4511)	(0.2931)	(0.5528)	(0.9803)	(0.8590)	(0.1216)		
Obs	5,172		3,225		1,947		5,172	
adj. *R*^2^	0.0824	0.0644	0.0927	0.0752	0.0679	0.0495		

a The column of (4) is the interaction effects between age factors and air quality.

Standard errors in parentheses, clustered at the Provincial level. ^*^, ^**^, and ^***^indicate significance at the levels of 10, 5, and 1%, respectively. The control variable results are not listed here.

**Table 5:** Heterogeneity of SRH and ADL among different regional groups of residents affected by air quality index.

	**Region**		**East**		**West**		**Central**		^a^ **Region1#AQI**		**Region2#AQI**	
	**(1)**		**(2)**		**(3)**		**(4)**		**(5)**		**(6)**	
	**SRH**	**ADL**	**SRH**	**ADL**	**SRH**	**ADL**	**SRH**	**ADL**	**SRH**	**ADL**	**SRH**	**ADL**
AQI	0.0821^**^	0.0532^**^	0.0515	0.1159^**^	0.0530	0.0533	−0.0011	0.2444^***^	−0.2157	0.1642	0.7942^*^	0.0068
	(0.0411)	(0.0133)	(0.6071)	(0.0262)	(0.5455)	(0.2489)	(0.9939)	(0.0013)	(0.6256)	(0.6027)	(0.0878)	(0.9836)
Cons	0.7909	0.7498^***^	1.2163	2.4510^***^	1.0771	−0.2679	1.3838	1.4921^**^				
	(0.1480)	(0.0086)	(0.3721)	(0.0008)	(0.4441)	(0.6956)	(0.1989)	(0.0108)				
Obs	5,172		1,774		1,783		1,615		5,172		5,172	
adj. *R^2^*	0.0847	0.0761	0.0952	0.0984	0.0628	0.0984	0.0869	0.0984				

a The columns of (5) and (6) are the interaction effects between regional factors and air quality.

Standard errors in parentheses, clustered at the Provincial level. ^*^, ^**^, and ^***^indicate significance at the levels of 10, 5, and 1%, respectively. The control variable results are not listed here.

**Table 6:** Heterogeneity of SRH and ADL among different gender groups of residents affected by air quality index.

			**Male**		**Female**		**^a^Sex#AQI**	
	**(1)**		**(2)**		**(3)**		**(4)**	
	**SRH**	**ADL**	**SRH**	**ADL**	**SRH**	**ADL**	**SRH**	**ADL**
AQI	0.0772^*^	0.0447^**^	0.1496^*^	0.1248^**^	0.0604	0.0300	0.3233	0.3622
	(0.0523)	(0.0370)	(0.0776)	(0.0248)	(0.1802)	(0.1805)	(0.3159)	(0.1618)
cons	0.7750	0.7545^***^	−1.6841	0.7114	1.3586^**^	0.7717^***^		
	(0.1575)	(0.0092)	(0.1661)	(0.3830)	(0.0265)	(0.0083)		
*Obs*	5172		1069		4103		5172	
adj. *R*^2^	0.0826	0.0530	0.1158	0.0660	0.0700	0.0289		

a The column of (4) is the interaction effects between sex factors and air quality.

Standard errors in parentheses, clustered at the Provincial level.

*, **, and *** indicate significance at the levels of 10, 5, and 1%, respectively. The control variable results are not listed here.

#### 3.3.2. Impact degree of exposure to six pollutants

Considering that different sources of pollutants have different degrees of health effects, the environmental variable was replaced by the average concentrations of PM_2.5_, PM_10_, SO_2_, NO_2_, CO, and O_3_ from 2017 to 2018 to test the robustness of the previous conclusions (13). According to Table 7, PM_10_ (*P* < 0.05), O_3_ (*P* < 0.01), and NO_2_ (*P* < 0.01) have significant effects on SRH of the elderly. According to the results in Table 8, PM_2.5_ (*P* < 0.05), PM_10_ (*P* < 0.01), CO (*P* < 0.05), O_3_ (*P* < 0.01), and NO_2_ (*P* < 0.01) have significant effects on the ADL of the elderly. After replacing variables, they still pass the significance test, indicating that the results of the previous model are reliable. Under the same conditions, in areas where O_3_ and NO_2_ are the main sources of pollutants, the residents are more vulnerable to health shocks.

**Table 7:** Results of SRH and pollutant heterogeneity analysis.

	**(1)**	**(2)**	**(3)**	**(4)**	**(5)**	**(6)**
	**SRH**	**SRH**	**SRH**	**SRH**	**SRH**	**SRH**
PM_2.5_	0.0030					
	(0.1468)					
PM_10_		0.0027^**^				
		(0.0240)				
SO_2_			0.0004			
			(0.9150)			
CO				0.0728		
				(0.5347)		
O_3_					0.0073^***^	
					(0.0018)	
NO_2_						0.0080^***^
						(0.0042)
Control	YES	YES	YES	YES	YES	YES

Standard errors in parentheses, clustered at the Provincial level. ^*^, ^**^, and ^***^indicate significance at the levels of 10, 5, and 1%, respectively.

**Table 8:** Results of ADL and pollutant heterogeneity analysis.

	**(1)**	**(2)**	**(3)**	**(4)**	**(5)**	**(6)**
	**ADL**	**ADL**	**ADL**	**ADL**	**ADL**	**ADL**
PM_2.5_	0.0070^**^					
	(0.0161)					
PM_10_		0.0045^***^				
		(0.0090)				
SO_2_			0.0046			
			(0.3587)			
CO				0.3763^**^		
				(0.0228)		
O_3_					0.0101^***^	
					(0.0031)	
NO_2_						0.0127^***^
						(0.0013)
Control	YES	YES	YES	YES	YES	YES
Cons	4.0699^*^	4.5990^**^	3.0791	2.8509	4.3806^**^	3.4585
	(0.0643)	(0.0395)	(0.1553)	(0.1845)	(0.0455)	(0.1066)

Standard errors in parentheses, clustered at the Provincial level. ^*^, ^**^, and ^***^indicate significance at the levels of 10, 5, and 1%, respectively.

## 4. Discussion

This paper used CHARLS2018 data, binary logit regression modeling, ordered logit regression modeling and stepwise regression to investigate the impact of AP on the health of the elderly. We took the annual average value of the AQI of the area where the elderly population was located as the proxy indicator to reflect long-term AP and divided the health impact into two dimensions: self-assessment of health and daily behavioral ability. AQI and six types of pollutants were included as independent variables, which not only shows the impact of different sources of pollutants on health shocks, but also supports the reliability of our results. The study found that AP had a positive effect on the occurrence of health shocks in older people. This was reflected in impaired ADL and poor SRH. It is worth noting that the effect of AP on health shocks in older people shows some heterogeneity, with the effect of AP on health shocks in older people being more pronounced in the 60–69 year age group, the eastern/central regions with better economic development, and in areas with high concentrations of NO_2_ and O_3_ in the air.

AP damages both physical and mental health (15–17), especially affecting the self-rated health of low socio-economic groups (13) and increasing the probability of ADL disability (18, 19). Previous studies concerning older age groups mostly concentrated on the effects on mortality but did not assess the extent to which AP affects negative health events among residents in an aging society. Our study, for the first time, examines the connection between air quality and its effects on health shocks in senior citizens. This study introduces the variable of “health shocks” and tries to combine early studies to measure the impact on health by using the deterioration of SRH and ADL. As an undefined concept, health shocks can be creatively defined as adverse occurrences that the elderly undergo with aging and degradation of physical function. Our analysis suggested that the air quality has an impact on their daily living ability as well as self-rated health, both of which are associated with an increase in health expenditure (44, 45). Therefore, the first marginal contribution of this study is to extend the horizon of research on health shocks in the context of aging. In addition, environmental issues are a major problem worldwide. Our study also enriches the systematic analyses of environmental pollution, providing fresh micro evidence for the impact of AP on the health of the elderly population.

Previous analyses of age and gender heterogeneity have not come to a consensus owing to the complexity of the source of contaminants (13, 18, 43). Compared with women, men are more likely to work outside and are susceptible to AP for a longer period of time (13), their health is thereby more negatively affected (43); nevertheless, female residents have the traits of a long life expectancy but greater inhalation of cooking fumes at home, which leads to ADL disability (18). Based on biophysical differences in vulnerability (age or baseline health) (46), it can be considered that people over 60 years old have a more significant and negative ADL disability (18). Interestingly, individuals facing the same environmental conditions show different health responses through defensive and compensatory behaviors in practical terms (47, 48). Elderly people over 70 are more likely to spend less time outdoors during periods of high pollution and choose to stay at home. As a result, the cumulative health damage is small in this age group, while older people between the ages of 60 and 70 years tend to work outside and provide intergenerational care (45, 49). Existing studies have categorized young-older people aged 60–69 years as a vulnerable group (50), but research on this group is insufficient, with most surveys reporting on the health of the entire older population. Different age groups have significantly different access to environmental resources and abilities to avoid environmental externalities (45); therefore, physical and mental health issues in this younger group should be explored in the future. Regional differences in the effects of AP on residents' health are significant in central China, which is consistent with previous studies (43, 51). However, we found that there is an internal difference in the air quality and ADL of the elderly in the central and western regions. This study updates the previous practice of dividing regions into two categories (East and Midwest) (51). According to the air quality data we studied, the average AQI in the eastern and central regions is 85 and 83, respectively, higher than 66 in the western regions. With regard to the significant difference in the east, a possible explanation is that the level of economic development in the east is higher than that in the west (35, 51), and the AP is serious, which has a more significant impact on the elderly exposed to pollutants. Based on data from the 2018 China Statistical Yearbook, 1,774 samples from the east show that the average per capita gross domestic product (GDP) is 79,065 Chinese Yuan (CNY), compared with 46,481 CNY in the western regions and 46,153 CNY in the central regions (28). Although the heterogeneity analysis was divided by geographical location, our result is similar to that grouped by GDP (18), and we have consistently concluded the significance of the high GDP group. Interestingly, Liu et al. (51) found the heterogeneity in the Midwest regions, but the health of the eastern population was not sensitive to AP (51). We suspect that the reason for the difference is that, on the one hand, we selected the samples in 2018 from CHARLS, while Liu et al. used the samples in 2015 from CHARLS (All data is available from https://charls.pku.edu.cn); on the other hand, we evaluated the deterioration of health, while the previous study reported the positive score of health status.

With regard to the significant impact path of pollution, our findings are consistent with previous studies. Briefly, NO_2_ and O_3_ proved to be more relevant to health (17, 52). NO_2_ is produced by vehicle emissions, petrochemical refineries, power plants and fuel combustion, and NO combines with O_3_ in the atmosphere to produce NO_2_ (53, 54). Deeply penetrating the lungs, NO can induce respiratory problems such as dyspnea, bronchospasm, and even pulmonary edema when inhaled in significant volumes (54–56). Concentrations exceeding 2.0 parts per million can affect T-lymphocytes, particularly CD8+ cells and NK cells that trigger immunological responses (56). Long-term exposure to high concentrations of NO_2_ leads to lung disease, impairs the sense of smell, and irritates the eye and nasal mucosa (53, 56). Ozone production is highly non-linearly related to volatile organic compounds (VOCs) and NOx. Ground-level ozone is produced through chemical reactions between NOx and natural sources (soil and rock weathering, volcanic ash, sea salt and biomass particles) or VOCs emitted by human activities (53, 55). Ozone exposure causes the formation of malondialdehyde in the mouse epidermis, depleting vitamins C and E and leading to skin disease (57). In addition, ozone has a low water solubility and enters the lungs deeply after inhalation (58). Over 3 years of tracking, major European cities reported daily ozone concentrations and daily death rates. The number of daily deaths, respiratory deaths, and cardiovascular deaths all increased in direct proportion to an increase in ozone concentration (59). Exposure to NO_2_ and O_3_ in air pollutants has led to an increase in emergency hospital admissions for cardiovascular and respiratory diseases (54, 55). However, most of the samples selected in our study were from rural areas, and the urbanization level is low, so the AP situation in cities was not well represented. Previous research included SO_2_, NO_2_ and PM_10_, pointing out that the concentration of SO_2_ and PM_10_ is the main factor affecting the disability of residents' ADL (18). In addition to physiological health, the increase of NO_2_ and O_3_ concentrations was also significantly related to depressive symptoms in the elderly (13, 17). We analyzed the six types of pollutants separately for supplementary demonstration. Hence, our second marginal contribution is to update the existing heterogeneity analysis on the impact of AP on health. Understanding the real extent of unexpected and serious health deterioration experienced by the elderly in the face of changes in the air quality at the individual level will also help local governments to formulate measures according to the heterogeneity and inequality of groups and regions.

Despite the practical relevance of the results of the analysis, there are some limitations to this study. First, the average value of AQI can empirically reflect the impact of long-term exposure to AP on residents' health status. It is undeniable that there are biases and fewer fine data compared with spatial data. Second, the matched samples were predominantly female and living in rural areas, which may affect the applicability and reliability of the findings to some extent. Third, as CHARLS2018 did not ask how long individuals had lived in their current residence, we failed to control for the residence time of the respondents. This problem can be solved by using CHARLS databases in 2015 and 2018, which is an important direction for further research. Finally, because limited by the sample data, the synergistic effects among pollutants are not explored in depth in this paper. By gradually addressing the above issues, we can further inform the government on air pollution management.

## 5. Conclusion

In this study, we found that air pollution has a significant positive impact on health shocks in old age. The government should focus on increasing efforts to combat environmental pollution and develop a long-term environmental health work plan. Second, the government should pay particular attention to the health status of older groups, especially those without pension support, in rural areas, and young-older people with low levels of education, who are among those at high risk of health shocks. The serious consequences of health shocks can be addressed by increasing the availability of pensions and spreading knowledge of environmental and health sciences. Finally, as young-older people are exposed to air pollution when working outside the home or caring for grandchildren, it is particularly important that they take the necessary measures to protect themselves to mitigate the health effects of air pollution.

## Data availability statement

Publicly available datasets were analyzed in this study. This data can be found here: https://charls.pku.edu.cn.

## Author contributions

XL and AX provided research ideas. XL contributed to data analysis and manuscript writing. YL and WD contributed to critical revisions of the manuscript. All authors have read and approved the final manuscript.

## References

[1] Song C Wu L Xie Y He J Chen X Wang T. Air pollution in China: status and spatiotemporal variations. Environ Pollut. (2017) 227:334–47. 10.1016/j.envpol.2017.04.075 28482313

[2] World Health Organization. World Health Statistics 2022: Monitoring Health for the Sdgs, Sustainable Development Goals: World Health Organization (2022). Available online at: http://www.who.int/publications/i/item/9789240051157 (accessed May 19, 2018).

[3] Zhang Q Crooks R. Toward an Environmentally Sustainable Future: Country Environmental Analysis of the People's Republic of China. Mandaluyong City, Philippines: Asian Development Bank (2012).

[4] National Bureau of Statistics. The Seventh National Population Census stats.gov.cn: National Bureau of Statistics. (2021). Available online at: http://www.stats.gov.cn/tjsj/zxfb/202105/t20210510_1817181.html (accessed May 11, 2021).

[5] Song S Chu GSF. Social security reform in China: the case of old-age insurance. Contemp Econ Policy. (1997) 15:85–93. 10.1111/j.1465-7287.1997.tb00468.x

[6] Agüero-Torres H von Strauss E Viitanen M Winblad B Fratiglioni L. Institutionalization in the elderly: the role of chronic diseases and dementia. Cross-sectional and longitudinal data from a population-based study. J Clin Epidemiol. (2001) 54:795–801. 10.1016/S0895-4356(00)00371-1 11470388

[7] Motl RW McAuley E. Physical activity, disability, and quality of life in older adults. Phys Med Rehabil Clin N Am. (2010) 21:299–308. 10.1016/j.pmr.2009.12.006 20494278

[8] Weuve J Kaufman JD Szpiro AA Curl C Puett RC Beck T. Exposure to traffic-related AP in relation to progression in physical disability among older adults. Environ Health Perspect. (2016) 124:1000–8. 10.1289/ehp.1510089 27022889 PMC4937863

[9] Bu X Xie Z Liu J Wei L Wang X Chen M. Global Pm2.5-attributable health burden from 1990 to 2017: estimates from the global burden of disease study 2017. Environ Res. (2021) 197:111123. 10.1016/j.envres.2021.111123 33823194

[10] Kettunen J Lanki T Tiittanen P Aalto PP Koskentalo T Kulmala M. Associations of fine and ultrafine particulate air pollution with stroke mortality in an area of low air pollution levels. Stroke. (2007) 38:918–22. 10.1161/01.STR.0000257999.49706.3b 17303767

[11] Hu K Keenan K Hale JM Börger T. The association between city-level air pollution and frailty among the elderly population in China. Health Place. (2020) 64:102362. 10.1016/j.healthplace.2020.102362 32838887

[12] Leung DYC. Outdoor-indoor air pollution in urban environment: challenges and opportunity. Front Environ Sci. (2015) 2:69. 10.3389/fenvs.2014.00069

[13] Shen WT Yu X Zhong SB Ge HR. Population health effects of air pollution: fresh evidence from china health and retirement longitudinal survey. Front Public Health. (2021) 9:779552. 10.3389/fpubh.2021.779552 35004584 PMC8733201

[14] Peled R. Air pollution exposure: who is at high risk? Atmos Environ. (2011) 45:1781–5. 10.1016/j.atmosenv.2011.01.001

[15] Giovanis E Ozdamar O. Health status, mental health and air quality: evidence from pensioners in Europe. Environ Sci Pollut Res. (2018) 25:14206–25. 10.1007/s11356-018-1534-0 29525857 PMC5978846

[16] Jiao K Xu M Liu M. Health status and air pollution related socioeconomic concerns in Urban China. Int J Equity Health. (2018) 17:1–11. 10.1186/s12939-018-0719-y 29402280 PMC5800084

[17] Lim YH Kim H Kim JH Bae S Park HY Hong YC. Air pollution and depressive symptoms in elderly adults. Environ Health Perspect. (2012) 120:1023–8. 10.1289/ehp.1104100 22514209 PMC3404652

[18] Liu H. Determining the effect of air quality on activities of daily living disability: using tracking survey data from 122 cities in China. BMC Public Health. (2022) 22:1–16. 10.1186/s12889-022-13240-7 35473502 PMC9044699

[19] Zeng Y Gu D Purser J Hoenig H Christakis N. Associations of environmental factors with elderly health and mortality in China. Am J Public Health. (2010) 100:298–305. 10.2105/AJPH.2008.154971 20019314 PMC2804639

[20] Li L Lin GZ Liu HZ Guo Y Ou CQ Chen PY. Can the air pollution index be used to communicate the health risks of air pollution? Environ Pollut. (2015) 205:153–60. 10.1016/j.envpol.2015.05.038 26057478

[21] Mehmood K Bao Y Abbas R. Saifullah Petropoulos GP Ahmad HR. Pollution characteristics and human health risk assessments of toxic metals and particle pollutants *via* soil and air using geoinformation in urbanized City of Pakistan. Environ Sci Pollut Res Int. (2021) 28:58206–20. 10.1007/s11356-021-14436-x 34110590

[22] Ministry of Ecology and Environment of the People's Republic of China. Technical Regulation on Ambient Air Quality Index. (on Trial): mee.gov.cn. (2016). Available online at: https://www.mee.gov.cn/ywgz/fgbz/bz/bzwb/jcffbz/201203/t20120302_224166.shtml (accessed January 1, 2016).

[23] Ministry of Ecology and Environment of the People's Republic of China. Ambient Air Quality Standards: mee.gov.cn. (2016). Available online at: https://www.mee.gov.cn/ywgz/fgbz/bz/bzwb/dqhjbh/dqhjzlbz/201203/t20120302_224165.shtml (accessed January 1, 2016).

[24] Aqistudy.cn. Historical Data of Pm2.5 | Historical Data of Air Quality Index | Historical Data of China Air Quality Online Monitoring and Analysis Platform aqistudy.cn. Available online at: https://www.aqistudy.cn/historydata/ (accessed July 01, 2017).

[25] Dong Q Wang Y Li P. Multifractal behavior of an air pollutant time series and the relevance to the predictability. Environ Pollut. (2017) 222:444–57. 10.1016/j.envpol.2016.11.090 28012664

[26] Wei Q Wu J Zhang Y Cheng Q Bai L Duan J. Short-term exposure to sulfur dioxide and the risk of childhood hand, foot, and mouth disease during different seasons in Hefei, China. Sci Total Environ. (2019) 658:116–21. 10.1016/j.scitotenv.2018.11.481 30577010

[27] Data Center of Ministry of Ecology Environment the People's Republic of China: mee.gov.cn. Available online at: https://www.mee.gov.cn/ (accessed December 20, 2018).

[28] National Bureau of Statistics. 2018 China Statistical Yearbook: China Statistics Press. (2018). Available online at: http://www.stats.gov.cn/tjsj/ndsj/2018/indexeh.htm (accessed October 12, 2018).

[29] Sundmacher L. The effect of health shocks on smoking and obesity. Eur J Health Econ. (2012) 13:451–60. 10.1007/s10198-011-0316-0 21559942

[30] García-Gómez P. Institutions, health shocks and labour market outcomes across Europe. J Health Econ. (2011) 30:200–13. 10.1016/j.jhealeco.2010.11.003 21167616

[31] Jones AM Rice N Roberts J. Sick of work or too sick to work? Evidence on self-reported health shocks and early retirement from the Bhps. Econ Modell. (2010) 27:866–80. 10.1016/j.econmod.2009.10.001

[32] Mitra S Palmer M Mont D Groce N. Can households cope with health shocks in Vietnam? Health Econ. (2016) 25:888–907. 10.1002/hec.3196 26017577 PMC4975721

[33] Wang X Sun M Li X Lu J Chen G. Effects of disability type on the association between age and non-communicable disease risk factors among elderly persons with disabilities in Shanghai, China. Int J Environ Res Public Health. (2020) 17:5426. 10.3390/ijerph17155426 32731459 PMC7432529

[34] Demura S Sato S Minami M. Utility of an adl index for institutionalized elderly people: examining possible applications for independent elderly people. Environ Health Prev Med. (2001) 6:33–40. 10.1007/BF02897307 21432235 PMC2723652

[35] Tang S Xu Y Li Z Yang T Qian D. Does economic support have an impact on the health status of elderly patients with chronic diseases in China?—Based on Charls. Data research. Front Public Health. (2021) 9:658830. 10.3389/fpubh.2021.658830 33959585 PMC8095396

[36] Grossman M. On the concept of health capital and the demand for health. J Political Econ. (1972) 80:223–55. 10.1086/259880

[37] Zeng Y Xu W Tao X. What factors are associated with utilisation of health services for the poor elderly? Evidence from a nationally representative longitudinal survey in China. BMJ Open. (2022) 12:e059758. 10.1136/bmjopen-2021-059758 35760535 PMC9237900

[38] Dastoorpoor M Sekhavatpour Z Masoumi K Mohammadi MJ Aghababaeian H Khanjani N. Air pollution and hospital admissions for cardiovascular diseases in Ahvaz, Iran. Sci Total Environ. (2019) 652:1318–30. 10.1016/j.scitotenv.2018.10.285 30586817

[39] Qin XD Qian Z Vaughn MG Trevathan E Emo B Paul G. Gender-specific differences of interaction between obesity and air pollution on stroke and cardiovascular diseases in chinese adults from a high pollution range area: a large population based cross sectional study. Sci Total Environ. (2015) 529:243–8. 10.1016/j.scitotenv.2015.05.041 26022408

[40] Nafstad P Håheim LL Oftedal B Gram F Holme I Hjermann I. Lung cancer and air pollution: a 27 year follow up of 16 209 Norwegian men. Thorax. (2003) 58:1071–6. 10.1136/thorax.58.12.1071 14645978 PMC1746548

[41] Greenberg N Carel RS Dubnov J Derazne E Portnov BA. Prevalence of asthma among young men residing in urban areas with different sources of air pollution. Isr Med Assoc J. (2019) 21:785–9. 31814340

[42] Dadvand P Nieuwenhuijsen MJ Esnaola M Forns J Basagaña X Alvarez-Pedrerol M. Green spaces and cognitive development in primary schoolchildren. Proc Nat Acad Sci. (2015) 112:7937–42. 10.1073/pnas.1503402112 26080420 PMC4491800

[43] Gu H Cao Y Elahi E Jha SK. Human health damages related to air pollution in China. Environ Sci Pollut Res. (2019) 26:13115–25. 10.1007/s11356-019-04708-y 30900129

[44] Chen F Chen Z. Cost of economic growth: air pollution and health expenditure. Sci Total Environ. (2021) 755:142543. 10.1016/j.scitotenv.2020.142543 33035980

[45] Wen M Gu D. Air Pollution shortens life expectancy and health expectancy for older adults: the case of China. J Gerontol A Biol Sci Med Sci. (2012) 67:1219–29. 10.1093/gerona/gls094 22518820

[46] Giaccherini M Kopinska J Palma A. When particulate matter strikes cities: social disparities and health costs of air pollution. J Health Econ. (2021) 78:102478. 10.1016/j.jhealeco.2021.102478 34161900

[47] Deschenes O Greenstone M Shapiro JS. Defensive investments and the demand for air quality: evidence from the nox budget program. Am Econ Rev. (2017) 107:2958–89. 10.1257/aer.20131002

[48] Graff Zivin J Neidell M. Environment, health, and human capital. J Econ Lit. (2013) 51:689–730. 10.1257/jel.51.3.689

[49] Sandström T Frew AJ Svartengren M Viegi G. The need for a focus on air pollution research in the elderly. Eur Respir J Suppl. (2003) 40:92s–5s. 10.1183/09031936.03.00403503 12762582

[50] Tang S Li T Fang J Chen R Cha Y Wang Y. The Exposome in practice: an exploratory panel study of biomarkers of air pollutant exposure in Chinese people aged 60–69 years. Environ Int. (2021) 157:106866. 10.1016/j.envint.2021.106866 34525388

[51] Liu W Xu Z Yang T. Health effects of air pollution in China. Int J Environ Res Public Health. (2018) 15:1471. 10.3390/ijerph15071471 30002305 PMC6068713

[52] Hossain MS Frey HC Louie PKK Lau AKH. Combined effects of increased o3 and reduced no 2 concentrations on short-term air pollution health risks in Hong Kong. Environ Pollut. (2021) 270:116280. 10.1016/j.envpol.2020.116280 33360064

[53] Hagenbjörk A Malmqvist E Mattisson K Sommar NJ Modig L. The Spatial Variation of O(3), No, No(2) and No. (X) and the Relation between them in two Swedish cities. Environ Monit Assess. (2017) 189:161. 10.1007/s10661-017-5872-z 28290139 PMC5348563

[54] Manisalidis I Stavropoulou E Stavropoulos A Bezirtzoglou E. Environmental and health impacts of air pollution: a review. Front Public Health. (2020) 8:14. 10.3389/fpubh.2020.00014 32154200 PMC7044178

[55] Zhang X Fung JCH Lau AKH Hossain MS Louie PKK Huang W. Air quality and synergistic health effects of ozone and nitrogen oxides in response to china's integrated air quality control policies during 2015–2019. Chemosphere. (2021) 268:129385. 10.1016/j.chemosphere.2020.129385 33383278

[56] Chen TM Gokhale J Shofer S Kuschner WG. Outdoor air pollution: nitrogen dioxide, sulfur dioxide, and carbon monoxide health effects. Am J Med Sci. (2007) 333:249–56. 10.1097/MAJ.0b013e31803b900f 17435420

[57] Thiele JJ Traber MG Tsang K Cross CE Packer L. *In vivo* exposure to ozone depletes vitamins C and E and induces lipid peroxidation in epidermal layers of murine skin. Free Radic Biol Med. (1997) 23:365–91. 10.1016/S0891-5849(96)00617-X 9214574

[58] Hatch GE Slade R Harris LP McDonnell WF Devlin RB Koren HS. Ozone dose and effect in humans and rats. A comparison using oxygen-18 labeling and bronchoalveolar lavage. Am J Respir Crit Care Med. (1994) 150:676–83. 10.1164/ajrccm.150.3.8087337 8087337

[59] Gryparis A Forsberg B Katsouyanni K Analitis A Touloumi G Schwartz J. Acute effects of ozone on mortality from the “air pollution and health: a European approach” project. Am J Respir Crit Care Med. (2004) 170:1080–7. 10.1164/rccm.200403-333OC 15282198

